# Biochemical analysis of collagens from the bone of lizardfish (*Saurida tumbil* Bloch, 1795) extracted with different acids

**DOI:** 10.7717/peerj.13103

**Published:** 2022-03-15

**Authors:** Abdul Aziz Jaziri, Rossita Shapawi, Ruzaidi Azli Mohd Mokhtar, Wan Norhana Md. Noordin, Nurul Huda

**Affiliations:** 1 Faculty of Food Science and Nutrition, Universiti Malaysia Sabah, Kota Kinabalu, Sabah, Malaysia; 2 Faculty of Fisheries and Marine Science, Universitas Brawijaya, Malang, East Java, Indonesia; 3 Borneo Marine Research Institute, Universiti Malaysia Sabah, Kota Kinabalu, Sabah, Malaysia; 4 Biotechnology Research Institute, Universiti Malaysia Sabah, Kota Kinabalu, Sabah, Malaysia; 5 Fisheries Research Institute, Batu Maung, Penang, Malaysia

**Keywords:** Acid-extracted collagen, Biochemical properties, Lizardfish by-products

## Abstract

**Background:**

Lizardfish (*Saurida tumbil* Bloch, 1795) bone is a fish by-product generated during industrial surimi processing. This by-product is an important source of collagen production since the use of terrestrial animal-based collagens no longer sought due to concern regarding the transfer of infectious diseases and religious issues. Hence, this study was carried out to determine the biochemical analysis of collagens from the bone of lizardfish extracted with different acids.

**Methods:**

Lizardfish bone collagens were extracted with various acids (*i.e.*, acetic, lactic and citric acids). All extraction processes were conducted in a chiller room (4 °C). The extracted collagens were biochemically characterized, such as hydroxyproline content, Ultraviolet (UV) absorption, X-ray diffraction (XRD), Fourier transform infrared spectroscopy spectra (FTIR), Differential scanning calorimetry (DSC) and solubility in different pH values and NaCl concentrations.

**Results:**

The yield of extracted collagens ranged between 1.73% and 2.59%, with the highest (*p* < 0.05) observed in citric acid-extracted collagen (CaEC). Protein patterns confirmed that all-collagen samples had two identical subunits, α1 and α2, representing type I collagen. The highest whiteness value was found in acetic acid-extracted collagen (AaEC), but there was no significant difference (*p* ≥ 0.05) compared to lactic acid-extracted collagen (LaEC). UV absorption and XRD analysis reflected the characteristics of the collagen, as reported in the literature. For the FTIR, all acid-extracted collagen samples presented a triple helical structure. The thermal transition temperature (*T*_max_ = 77.92–89.04 °C) was in accordance with collagen extracted from other fish species. All extracted collagens were highly soluble in acidic pH and low concentrations of NaCl (0–20 g/L). In conclusion, collagens extracted from lizardfish bone may be used as alternative sources of collagen in industrial settings, and AaEC would be considered superior in terms of the characteristics evaluated in this study.

## Introduction

Lizardfish (*Saurida tumbil* Bloch, 1795), belonging to the family Synodantidae, is typically characterized by cylindrical bodies and heads that superficially resemble those of lizards. It is an economically important fish species for surimi production in Malaysia, owing to its strong gel-forming capacity, white flesh color and high quantity of muscle (Itoh et al., 1995). In addition, this tropical species is affordable and commonly available. According to the Department of Fisheries Malaysia (The Department of Fisheries Malaysia, 2021), the average production of lizardfish from 2015 to 2019 was approximately 48,153 metric tons and was mostly used as a raw material for surimi processing. During surimi processing, a large quantity of fish by-products (viz. head, bone, viscera, skin, fins and scale) is generated, amounting to approximately 60–75% of the whole fish weight. The by-products are often utilized as animal feed, silage, fish meal and fertilizer, which are categorized as low value-added products (Nawaz et al., 2020). On the other hand, underutilized/nonutilized by-products are discarded with or without treatments into landfill sites and/or aquatic bodies. Because of the abundant organic matter found in the by-products, the waste should be treated prior to disposal. This activity, however, imposes a financial burden on seafood processors (Jayathilakan et al., 2012). Efforts are needed to convert fish by-products into valuable goods with high value not only as potential extra revenue but also to reduce environmental problems. Fish collagen is one of the high value products that can be extracted from surimi by-products.

Collagen is a fibrous protein that provides strength and structure in various connective tissues of animals, including bones, skin, ligaments and tendons. It is the most abundant protein, accounting for approximately one-third of the protein composition (León-López et al., 2019). To date, approximately 29 types of collagens have been documented in different tissues, and each type is classified based on a unique protein structure and amino acid sequence (Sorushanova et al., 2019). Among the types studied, type I collagen is the most predominant collagen observed in bones and skin tissue (Salvatore et al., 2020). In the industrial setting, type I collagen is extensively used in food and beverages, cosmetics, pharmaceuticals and health care (Hashim et al., 2015). Interestingly, the global market of collagen was estimated at 936.5 tons in 2020 and is projected to increase at approximately 5.6% due to an increase in health-awareness consumer preferences (GMI, 2021). In general, collagen derived from land-based animals such as cows, pigs and chickens’ bone and skin are excellent sources for collagen production. However, the use of animal-based collagens is no longer sought due to concern regarding the transfer of infectious diseases, such as bovine spongiform encephalopathy, foot-and-mouth disease and avian influenza. In addition, some religions, such as Hindus and Sikhs, prohibit the usage of bovine-based products, while porcine-originated collagen is not permissible for Muslims and Jews (Coppola et al., 2020; Lim et al., 2019; Jaziri, Muyasyaroh & Firdaus, 2020). As an alternative, in recent years, several studies have been drawn on fish collagen. Fish collagen has similar and even better characteristics than collagen derived from terrestrial animals after being modified its structure (He et al., 2011; Zhang et al., 2020).

The extraction process plays an essential role in obtaining collagen from fish by-products. The extraction of fish collagen could be employed using acid-, pepsin- and ultrasound-assisted procedures (Ahmed, Haq & Chun, 2019). However, acid-aiding procedures are preferred due to their lower cost, ease of control and greater practicality (Jongjareonrak et al., 2005). Organic acids, including acetic acid, lactic acid and citric acid, are usually used for collagen extraction (Tan & Chang, 2018). These acids are more effective in solubilizing non collagen chains and internal chains of collagen molecules than inorganic acids, such as hydrochloric acid (HCl) (Skierka & Sadowska, 2007). Moreover, the use of organic acids for fish collagen treatment could increase the yield of collagen in comparison with nonorganic acids (Schmidt et al., 2016). Numerous researchers have demonstrated collagen extraction using acid from a variety of fish species, such as bigeye snapper (Kittiphattanabawon et al., 2005), sailfish (Tamilmozhi, Veeruraj & Arumugam, 2013), grass carp (Wang et al., 2014), tilapia (Chen et al., 2016), puffer fish (Iswariya, Velswamy & Uma, 2018), barramundi (Liao et al., 2018), channel catfish (Tan & Chang, 2018), golden pompano (Cao et al., 2019), bigeye tuna (Ahmed, Verma & Patel, 2020), red stingray (Chen et al., 2019), giant grouper (Upasen et al., 2019) and sturgeon fish (Atef et al., 2020). Although acetic acid is often used, other organic acids could also be applied in collagen extraction.

Utilization of lizardfish bone is an effort to increase the added value of by-products. Around 17.60% of lizardfish by-products was bone portion and about 17.96% of its chemical composition was protein (based on wet basis) (Jaziri et al., 2021). A previous study from Taheri et al. (2009) has documented that lizardfish (*S. tumbil*) skin and bone could be utilized for gelatin. However, study on collagen from lizardfish bone is much less investigated to date. Therefore, the present study aimed to extract collagen from the bone of lizardfish from tropical marine waters using different acids (acetic, lactic and citric acid). Biochemical properties were also evaluated.

## Materials & Methods

### Chemicals

Commercial fish collagen hydrolysate (type I collagen) was purchased from SRB Suria Trading (Kedah, Malaysia). Coomassie Blue R-250 (C.I. 42660), sodium dodecyl sulfate (SDS) (8170341000), N,N,N’,N’-tetramethyl ethylene diamine (TEMED) (1107320100), acrylamide (8008300500), Lowry reagent (L3540-25 VL), Folin-Ciocalteu’s phenol reagent (1090010100) and acetic acid (1000632511) were obtained from Merck (Darmstadt, Germany). Lactic acid (CAS 79-33-4) was bought from Bendosen (Kuala Lumpur, Malaysia) and citric acid (C1050-90) was obtained from Systerm (Selangor, Malaysia). Tris(hydroxymethyl) aminomethane hydrochloride (CAS 1185-53-1) and bovine serum albumin (BSA) (A2153-10G) were supplied by the Sigma Chemical Co. (St. Louis, MO, USA). Molecular weight markers using prestained natural protein standards (dual color standards) (1610374) was purchased from BioRad Laboratories (Hercules, CA, USA). Other chemicals used in this study were of analytical grade.

### Preparation of lizardfish bone

Fresh lizardfish (*S. tumbil*) was purchased from a wet market in Kota Kinabalu, Sabah, Malaysia. Samples were placed in an insulated box containing ice at a ratio of fish/ice was 1:2 (w/w) to keep their freshness and delivered to the laboratory within 20 min. After species identification and authentication, the fish samples were weighed (202.58 ± 16.05 g), and the total length was measured (29.86 ± 0.41). The fish was washed with running tap water and subjected to separation of flesh, head, bone, skin and fins using a mechanical deboner machine (SFD-8, Taiwan). For the lizardfish bone collected, an approximate 1.0 × 1.0 cm^2^ sample size was prepared for collagen extraction. Prepared fish bone samples were washed with chilled water and then placed in polyethylene containers. The packed samples were kept in a freezer (−20 °C) until further analysis.

### Extraction of acid-soluble collagens

**Figure 1:**
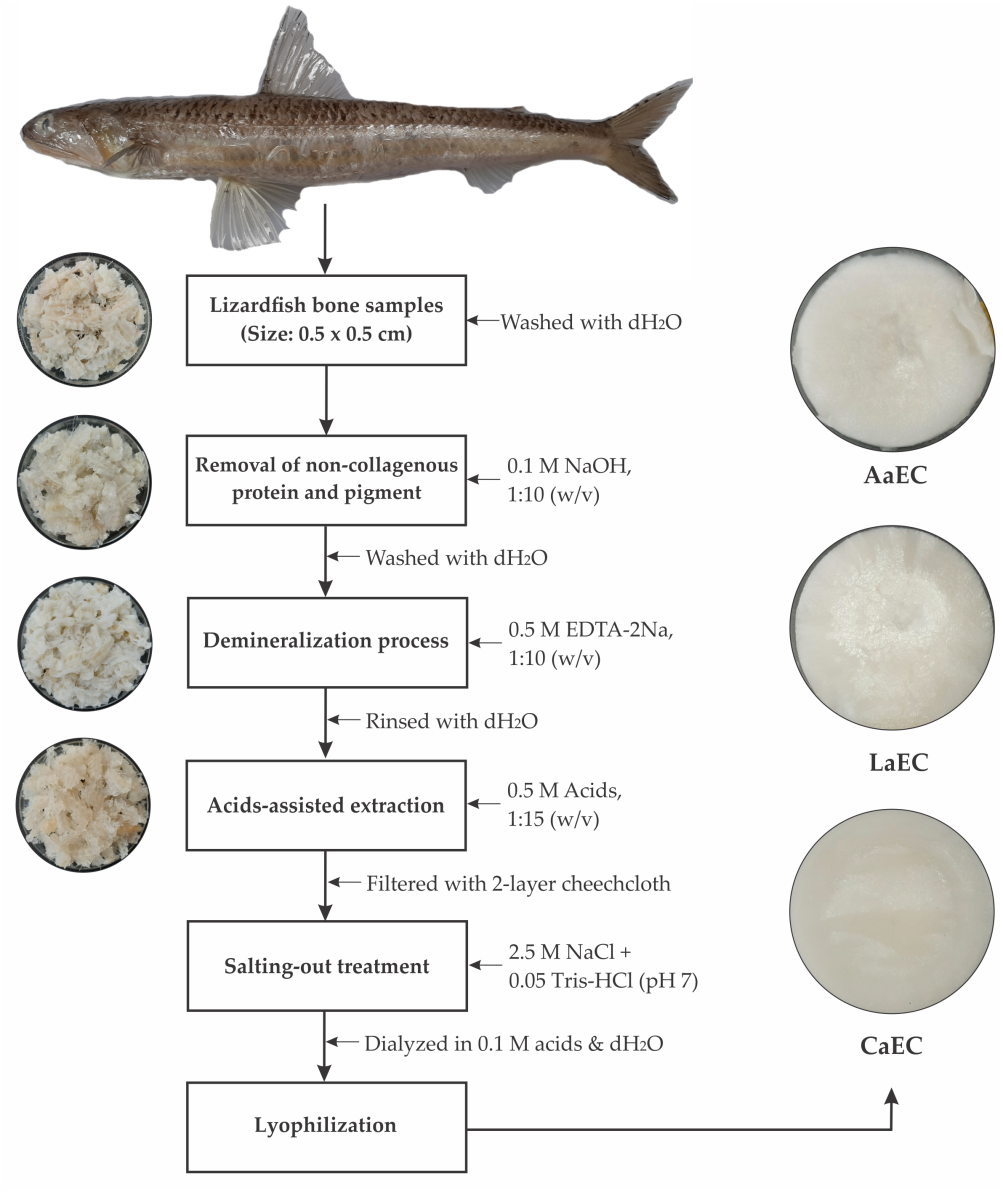
Acid extraction process of collagen derived from the bone of lizardfish. AaEC, Acetic acid-extracted collagen; LaEC, Lactic acid-extracted collagen; CaEC, Citric acid-extracted collagen.

Acid-soluble collagens (ASCs) were carried out according to the method published by Matmaroh et al. (2011) with modifications in demineralization, extraction and centrifugation time. All procedures employed in this extraction process were performed in a chiller (4 °C), and the process is shown in Fig. 1. Thawed fish bones were dissolved with 0.1 M NaOH at a ratio of 1:10 (w/v) for 6 h to remove pigments and non-collagenous proteins, and the solution was changed every 3 h. The treated samples were then washed with cold distilled water until pH 7 was achieved. For the demineralization process, neutral pH samples were added to 0.5 EDTA-2Na solution at pH 7.4 for 48 h at a ratio of 1:10 (w/v), and the solution was changed every 16 h. Next, demineralized samples were washed with cold distilled water three times with continuous stirring for 10 min. and the samples were extracted with different acid solutions, *i.e.,* acetic acid, lactic acid and citric acid, with 0.5 M of each acid used. Acid extraction was conducted by adding acids at a ratio of 1:15 (w/v) for 72 h with continuous stirring. After extraction, treated samples were filtered through 2 layers of cheese cloth. The filtrates were then collected and salted out by adding 2.5 M of NaCl containing 0.05 M Tris (hydroxymethyl) aminomethane (pH 7). Afterwards, the salted-out samples were centrifuged at 15,000× g for 30 min, and the pellets were dissolved in 0.5 M acids at a ratio of 1:5 (w/v). The solubilized samples were dialyzed using a dialysis tubing cellulose membrane (flat width 43 mm, Sigma) in a 20 volume of 0.1 M acetic, lactic and citric acids, respectively for 24 h and the solutions was changed with distilled water for 48 h. The dialyzed samples were lyophilized using a freeze-dryer (Labconco, South Kansas City, KS, USA). ASCs from different acid treatments were then stored in a freezer (−20 °C) until use. For measuring yield, a formula was adopted from Benjakul et al. (2010) based on the initial weight of lyophilized collagen in comparison with the initial weight of fish bone. For hydroxyproline content, all extracted collagens were analyzed using the method developed by Hofman et al. (2011).

### Collagen analyses

### Color attributes

Color ASCs and commercial fish collagen hydrolysates (CoFC) were determined according to the method reported by Huda et al. (2013) using a colorimeter model ColorFlex CX2379 (HunterLab, Reston, VA, USA). Parameters of color were tested, including lightness (L*), redness (a*) and yellowness (b*). Approximately 500 mg of fiber collagens was placed into a clear glass cup and then inserted into a cell holder of a colorimeter. The whiteness index (WI) was determined according to the study of Briones & Aguilera (2005) using the following formula:

\begin{eqnarray*}\text{WI}=100-[(100-{L}^{\ast })^{2}+ \left( {a}^{\ast 2} \right) +({b}^{\ast 2})]^{0.5}. \end{eqnarray*}

### Sodium dodecyl sulfate-polyacrylamide gel electrophoresis

Sodium dodecyl sulfate-polyacrylamide gel electrophoresis (SDS–PAGE) of all ASC and standard samples was performed according to the method from Laemmli (1970) with slight modifications. Approximately 2.5 mg of collagen was dissolved in one mL of SDS (5%), and the mixtures were heated at 85 °C for 1 h in a water bath. Afterward, the heated samples were centrifuged at 8,500× g for 5 min at 25 °C. The supernatants were pipetted and transferred into a new centrifuge tube. The prepared sample buffers (with and without 10% β-mercaptoethanol) were mixed with extracted collagens at a ratio of 1:1 (v/v) and then heated at 85 °C for 3 min. After thermal incubation, around 15 µL of the mixture was loaded into an acrylamide gel containing 5% stacking gel and 12% resolving gel. Next, electrophoresis was performed at a constant voltage of 120 V/gel for approximately 3 h using a Mini Protein II unit (Bio-Rad laboratories, Hercules, CA, USA). Acrylamide gels containing proteins were stained with 0.1% (w/v) Coomassie blue R-250 in 30% (v/v) methanol and 10% (v/v) acetic acid and destained with 30% (v/v) methanol and 10% (v/v) acetic acid. The molecular weight markers were determined using a dual color protein standard (10–250 kDa).

### UV absorption spectrum

All ASCs and control sample were scanned based on UV absorption spectra at wavelengths from 400 nm to 200 nm using a UV-Vis spectrophotometer (Agilent Cary 60; Agilent, Santa Clara, CA, USA). This procedure was adopted from Liao et al. (2018). Approximately 10 mg of each lizardfish collagen was dissolved in 0.5 M acetic acid at a ratio of 1:1 (w/v), and the solution was dropped into a quartz cell. The spectral data was directly recorded according to the wavenumber set. The baseline was set with 0.5 M acetic acid solution.

### Fourier transform infrared spectroscopy

Fourier transform infrared spectroscopy (FTIR) of the lyophilized collagen and CoFC samples was performed using a total reflectance-Fourier transform infrared (ATR-FTIR) (Agilent Cary 630, US) spectrometer according to the protocol from Matmaroh et al. (2011) with modification. One milligram of collagen was placed on a crystal cell. The spectra were adjusted within the wavenumbers of 4,000–600 cm^−1^ with a resolution of 2 cm^−1^ for 32 scans against a background spectrum recorded from the clean empty cells at room temperature. The resulting spectra were evaluated using the Agilent Microlab software program.

### X-ray diffraction

X-ray diffraction (XRD) of the extracted lizardfish bone collagens with three different acids and the collagen control was carried out using the method of Liao et al. (2018). The lyophilized collagens were scanned using an XRD instrument (Rigaku Smart Lab®, Japan) with copper Kα as a source of X-rays. The tube voltage and current were set at 40 kV and 40 mA, respectively. The scanning range was determined to be between 10° and 50° (2*θ*) with a speed of 0.06° per second.

### Differential scanning colorimetry

Determination of differential scanning colorimetry (DSC) in the lizardfish bone collagens and CoFC was conducted following the method of Sionkowska et al. (2015) using a DSC Q200 (TA Instruments, New Castle, DE, USA). The lyophilized collagens were rehydrated with deionized water at a ratio of 1:40 (w/v). The mixtures were allowed to stand for 2 days in a chiller (4 °C). Before scanning, the samples (3-10 mg) were precisely weighed into aluminum sample pans (PerkinElmer, Waltham, MA, USA) and tightly sealed. The sealed samples were heated from 20 °C to 250 °C in a nitrogen (N_2_) stream with a heating rate of 10 °C/min. An empty pan was prepared as the reference. The maximum transition temperature (*T*_max_) was measured from the endothermic peak of the thermogram, while the total denaturation enthalpy (Δ*H*) was determined by measuring the area of the DSC thermogram.

### Solubility evaluation

Solubility profile of collagens was tested with different pH and NaCl concentration prepared according to the methods of Thuy, Okazaki & Osako (2014). In terms of pH treatment, the prepared samples were dissolved in 0.5 M acetic acid solution with continuous stirring at 4 °C for 18 h. The solubilized collagens were then adjusted at different pH values (pH 1-11) with 1 N NaOH and HCl. Each solubility test was prepared approximately eight mL of solubilized samples with a concentration of 3 mg/mL. Distilled water was added to the solutions to obtain a final volume of 10 mL. Next, the collagen solutions were centrifuged at 15,000 rpm for 30 min at 4 °C. For NaCl treatment, five milliliters of solubilized samples were mixed with five mL of NaCl solution. The NaCl concentrations used in this study were in the range of 0–60 g/L. The mixtures were then stirred at 4 °C for 1 h using a magnetic stirrer. Then, the mixtures were centrifuged at 15,000 rpm for 30 min at 4 °C. Protein content in the supernatant was determined by the Lowry method (Lowry et al., 1951) using bovine serum albumin (BSA) as a standard. Relative solubility was calculated using the following equations:

\begin{eqnarray*}\text{Relative solubility}~ \left( \text{%} \right) = \frac{\text{Current concentration of protein}}{\text{The highest concentration of protein}} \times 100. \end{eqnarray*}

### Statistical analysis

All experiments were performed in triplicate, and the data are expressed as the means ± standard deviation. One way analysis of variance was performed, and mean comparisons were analyzed by Duncan’s multiple range tests using SPSS Statistics version 27.0 (IBM Corp., Armonk, New York).

## Results

### Yield and hydroxyproline content

**Figure 2:**
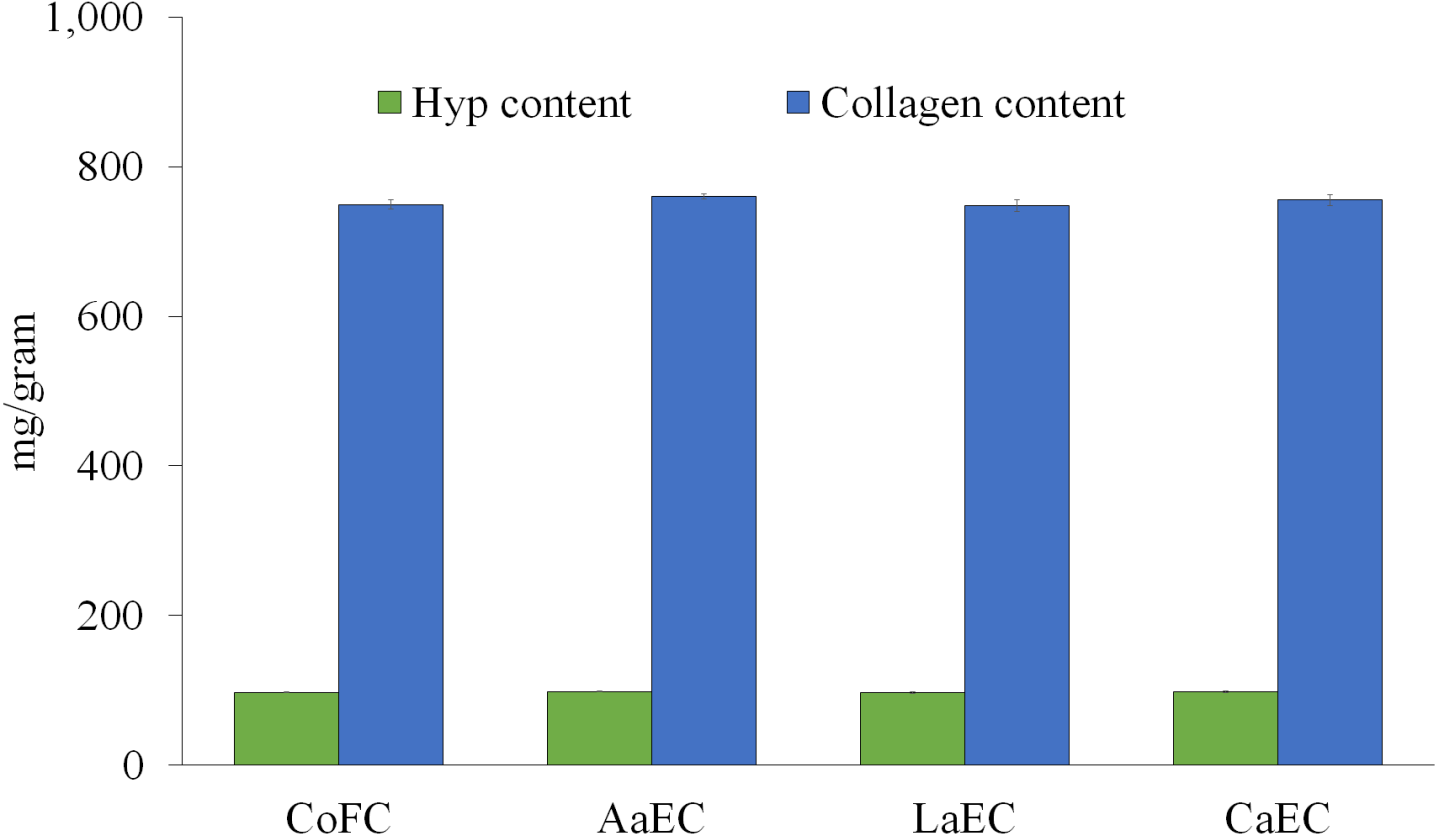
Hydroxyproline (Hyp) and collagen content of lizardfish bone collagen extracted with different acids. CoFC, commercial fish collagen hydrolysate (type I), acetic acid-extracted collagen; LaEC, lactic acid-extracted collagen; CaEC, citric acid-extracted collagen. Different letters in the same graph indicate significant differences (*P* < 0.05).

Collagen from lizardfish bone was extracted using three different acids (*i.e.,* acetic acid, lactic acid, and citric acid), the yields of collagen samples are presented at Table 1. The results showed a significant difference (*P* < 0.05) in the yields of collagen, *i.e.,* 1.73%, 1.88%, and 2.59% (based on wet weight of bone), for acetic acid, lactic acid, and citric acid, respectively. The total collagen (mg/g) could be quantified indirectly through hydroxyproline (Hyp) content, as this amino acid is present almost exclusively in collagen. The content of Hyp was initially determined in all extracted samples, and the total collagen was subsequently multiplied by 7.7 as a conversion factor according to the method of Kittiphattanabawon et al. (2005). The results obtained showed that acetic acid-extracted collagen (AaEC) had the highest content of Hyp (98.70 mg/g) compared with lactic acid-extracted collagen (LaEC) (97.10 mg/g) and citric acid-extracted collagen (CaEC) (98.10 mg/g). The AaEC was also higher than that reported from commercial fish collagen hysrolysate (CoFC) (97.30 mg/g). However, those collagen samples were not significantly different (*P* ≥ 0.05) (Fig. 2). The total collagen of AaEC, LaEC, CaEC and CoFC was 760 mg/g, 747.80 mg/g, 755.20 mg/g and 749.40 mg/g, respectively, and they were not significantly (*P* ≥ 0.05) different.

**Table 1:** Yield and colour attributes (*L*^∗^, *a*^∗^, *b*^∗^ and whiteness index) of collagens from lizardfish bones extracted with different acids.

Acids	Yield (%)	L*	a*	b*	Whiteness
CoFC	–	84.65 ± 0.57^a^	0.57 ± 0.03^b^	17.40 ± 0.41^d^	76.79 ± 0.21^a^
AaEC	1.73 ± 0.08^a^	88.54 ± 0.42^b^	0.20 ± 0.09^a^	9.23 ± 0.51^a^	85.29 ± 0.10^c^
LaEC	1.88 ± 0.03^b^	88.00 ± 0.45^b^	0.54 ± 0.02^b^	10.26 ± 0.10^b^	84.20 ± 0.35^bc^
CaEC	2.59 ± 0.52^c^	88.43 ± 0.94^b^	0.71 ± 0.10^b^	11.97 ± 0.35^c^	83.34 ± 0.89^b^

**Notes.**

Values are given as the mean ± standard deviation from triplicate determinations (*n* = 3). Different superscript letters in the same row indicate significant differences (*p* < 0.05).

CoFC commercial fish collagen hydrolysate (type I) AaEC acetic acid-extracted collagen LaEC lactic acid-extracted collagen CaEC citric acid-extracted collagen

### Color parameters

Color is one of the most important aspects of collagen as an ingredient in cosmetics, pharmaceuticals and particularly for food production (Sliburyte & Skeryte, 2014). Table 1 shows the color attributes of acid-soluble collagens (ASCs) extracted from lizardfish bone. All ASCs had significantly higher (*P* < 0.05) L* (lightness) values than commercial fish collagen. Although not significantly different (*P* ≥ 0.05), the lightness level of AaEC was the highest. On the other hand, the AaEC a* and b* values (*P* < 0.05) were significantly lower than those of LaEC, CaEC and even CoFC. For the whiteness index (*WI*), AaEC exhibited the highest (*P* < 0.05) value compared to LaEC, CaEC and CoFC.

### Protein profile

**Figure 3:**
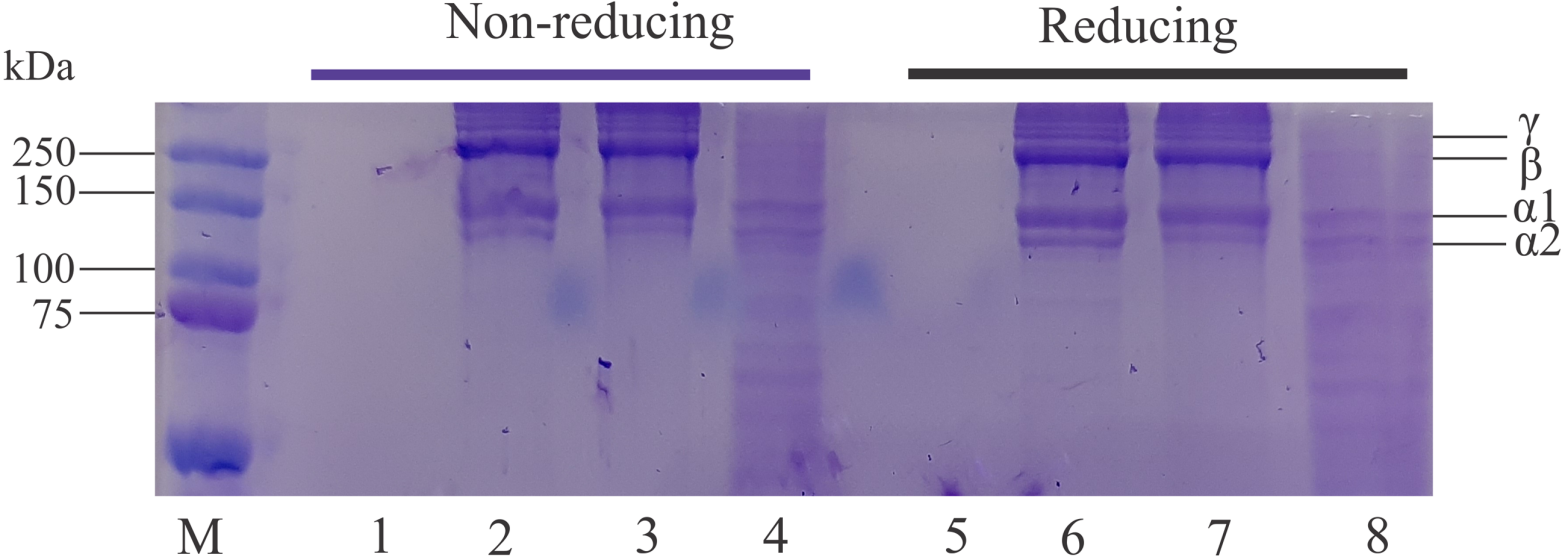
Protein pattern for acid-soluble collagen under nonreducing and reducing conditions. Lane M, molecular weight marker; Lanes 1 and 5, commercial fish collagen hydrolysate; Lane 2 and 6, acetic acid extraction; Lanes 3 and 7, lactic acid extraction; Lanes 4 and 8, citric acid extraction.

The protein profiles of lizardfish bone collagen extracted with different acids and CoFC sample under nonreducing and reducing conditions are displayed in Fig. 3. The results indicate that all extracted collagen samples contain two different α (α_1_ and α_2_) chains. The band intensity of α_1_ was higher than that of the α_2_ chain. Moreover, the intensity of bands in the LaEC and AaEC was greater than that in the CaEC with the same concentration applied. The estimated molecular weights of lizardfish collagens were 126.96 kDa (α_2_), 160.81 kDa (α_1_), 238.47 kDa (β-chain) and 290.39 kDa (*γ* chain). Furthermore, the effect of beta-mercaptoethanol (β-ME) (reducing) and no *β*-ME (non-reducing) additions exhibited similar electrophoretic patterns to all the extracted lizardfish bone collagens. On the other hand, there are no bands found in the commercial fish collagen hydrolysate.

### UV absorption spectrum

**Figure 4:**
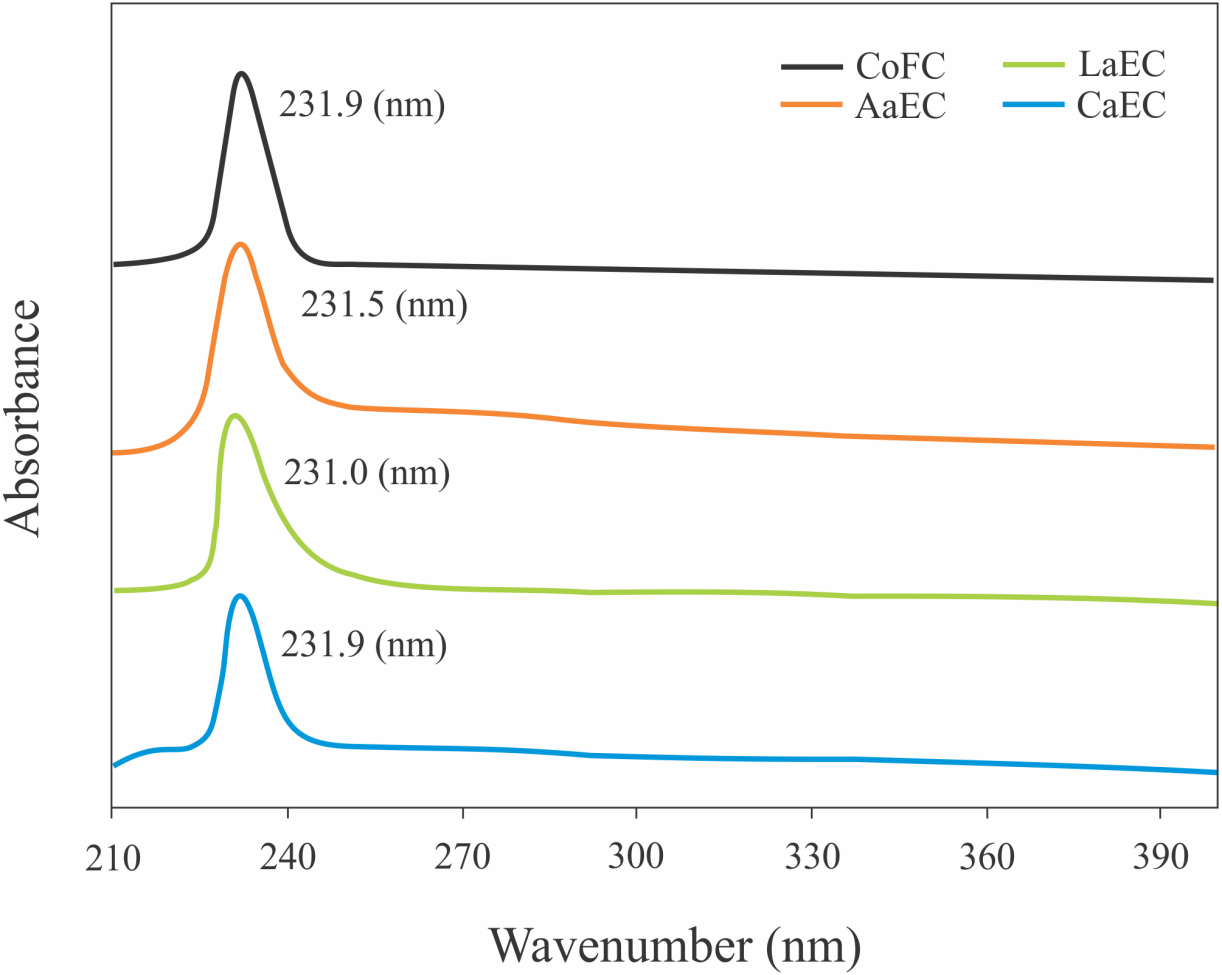
UV absorption of lizardfish bone collagens extracted with different acids. CoFC, Commercial fish collagen hydrolysate; AaEC, Acetic acid-extracted collagen; LaEC, Lactic acid-extracted collagen; CaEC, Citric acid-extracted collagen.

In general, the triple helical collagen has the characteristic absorption peak at approximately 230 nm (Liao et al., 2018). Figure 4 shows the maximum absorption of lizardfish bone collagens and commercial fish collagen. All the extracted samples (AaEC, LaEC and CaEC) and the CoFC sample had UV maximum absorptions in the range between 231.0 nm and 231.9 nm.

### FT-IR

**Figure 5:**
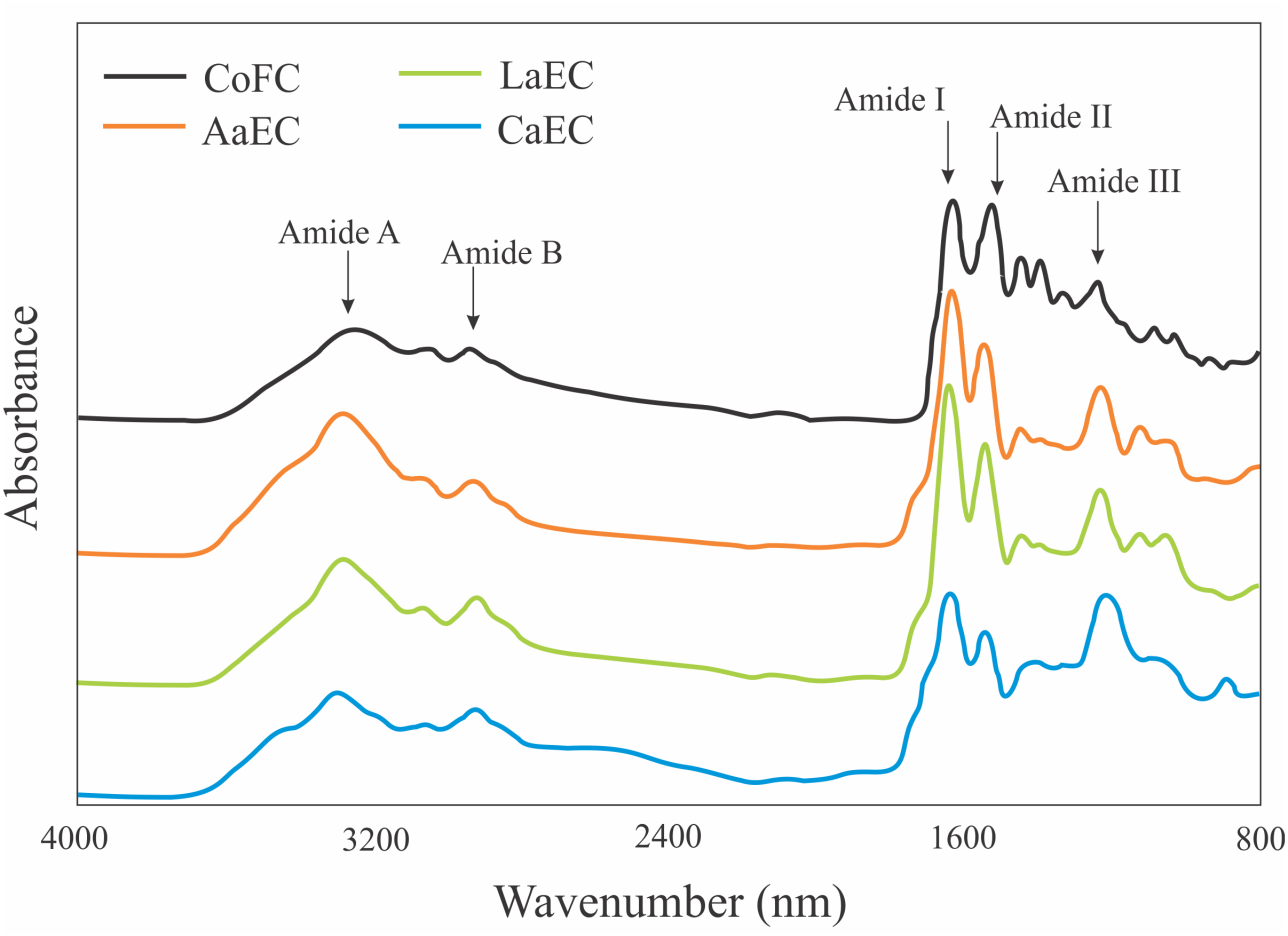
FTIR patterns of lizardfish bone collagen extracted with different acids. CoFC, Commercial fish collagen hydrolysate; AaEC, Acetic acid-extracted collagen; LaEC, Lactic acid-extracted collagen; CaEC, Citric acid-extracted collagen.

FT-IR spectra peaks and their assignments for the lizardfish bone collagens extracted with acetic, lactic, and citric acids are shown in Fig. 5. The peaks were identified as amide A, amide B and amides I, II, and III (Ahmed, Verma & Patel, 2020). The amide A band reflects N-H stretching paired with H bonds, and several peaks for the acid-extracted samples (AaEC, CaEC, and LaEC) ranged from 3289.46 cm^−1^ to 3295.05 cm^−1^, while the commercial fish collagen (CoFC) sample was characterized by the lowest wavenumber for the amide A attribute. The amide B peak positions of CoFC, AaEC, CaEC, and LaEC ranged between 2924.17 cm^−1^ and 2942.81 cm^−1^, which represented CH_2−_, CH_3−_, and C-H-asymmetric stretching. For amide I-III bands, peaks were obtained below 1650 cm^−1^ for all collagen samples.

### XRD

**Figure 6:**
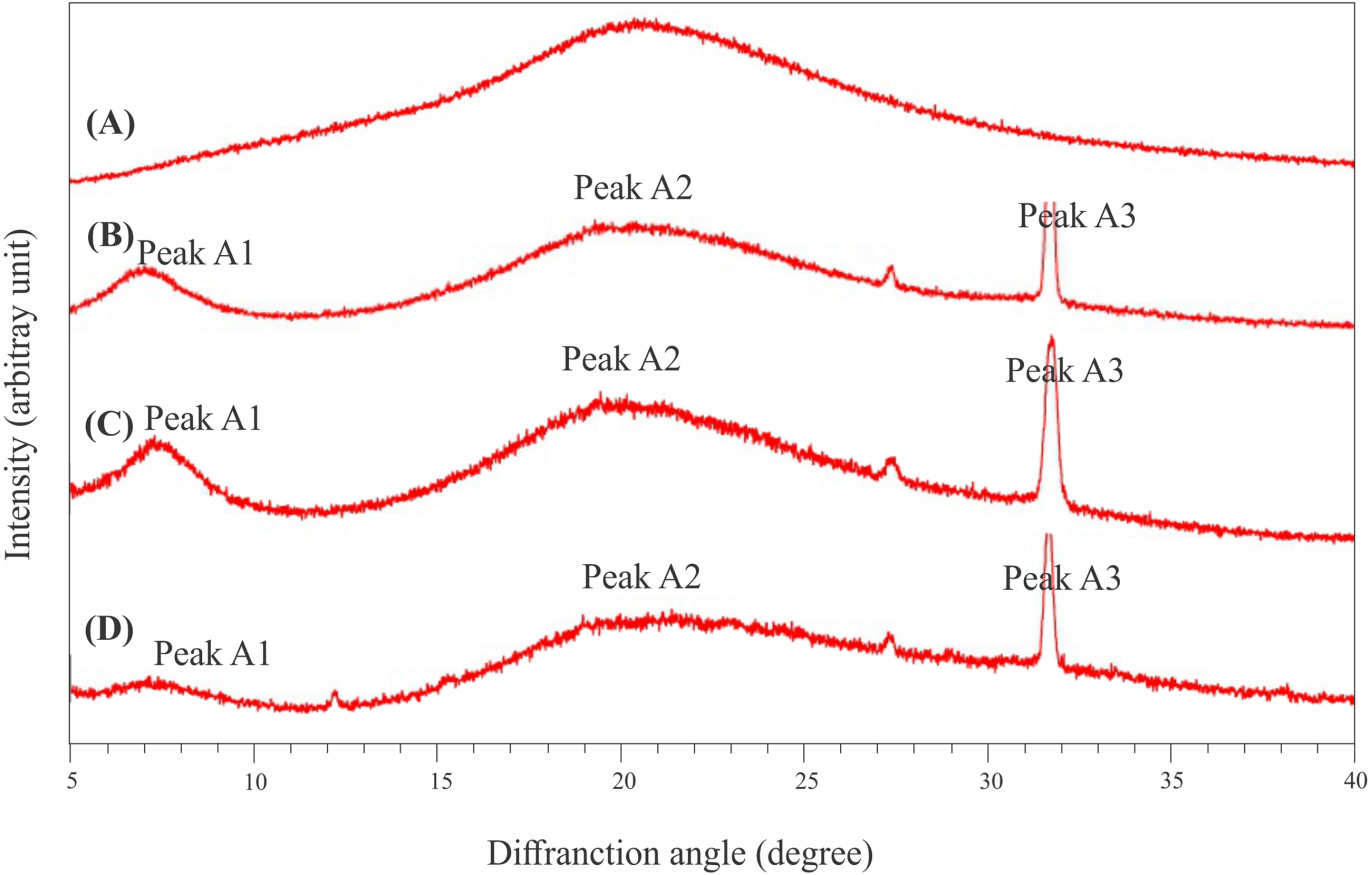
X-ray diffraction diagram of lizardfish bone collagens extracted with different acids and commercial fish collagen hydrolysate. (A) CoFC, commercial fish collagen hydrolysate, (B) AaEC, acetic acid-extracted collagen, (C) LaEC, lactic acid-extracted collagen and (D) CaEC, citric acid-extracted collagen.

All extracted collagen samples showed three diffraction peaks, while one diffraction peak was found in the CoFC sample (Fig. 6). The Bragg 2dsin*θ* = *λ* (*λ* = 0.154 Å) formula was employed to obtain the XRD results. The XRD patterns of AaEC, LaEC and CaEC had diffraction peaks at approximately 7.04°–32.71°, 7.41°–31.74°, and 7.15°–31.70°, respectively. Meanwhile, the commercial fish collagen hydrolysate harbored a diffraction peak at 20.52°. For ASCs, the first diffraction peak (A1) found in this study ranged between 7.04° and 7.41° with *d* values recorded from 12.49 Å to 12.50 Å. The CaEC sample recorded the greatest d value compared to the LaEC and AaEC samples. The second diffraction peak (A2) for all collagens was located between 19.54° and 21.32° with the CaEC sample showing the smallest *d* value (4.29 Å) compared with that of LaEC (5.01 Å), AaEC (4.40 Å) and CoFC (4.32 Å). The last diffraction peak, labeled A3, was observed in the range of 31.70°–31.74° with a similar d value (2.82 Å).

### Thermal stability

The thermal stability of lizardfish bone collagens and commercial fish collagen hydrolysates was determined using differential scanning calorimetry (DSC). Theoretically, the heat flow was measured between the sample and reference and subsequently provides information regarding the thermal transitions of proteins. The transition temperature value from all collagen samples was determined at the maximum transition point (*T*_max_) or the endothermic peak of transition curves, as presented in Table 2. All extracted collagens (AaEC, LaEC and CaEC) exhibited two noticeable peaks, and the first peak harbored *T*_max_ values of approximately 77.92–89.04 °C, which is associated with the release of the water bound in the collagen molecule as well as the degradation of the triple helical structure. Meanwhile, the second peak varied between 174.35 °C and 212.11 °C. This peak presents the melting temperature of the crosslinked collagen parts. In addition to the endothermic peak values, the denaturation enthalpy of the first transformation (Δ*H* = 169–294.5 J/g) was much higher compared to the second conversion (Δ*H* = 4.69–91.18 J/g).

**Table 2:** Thermal parameters of collagen extracted from lizardfish bone with different acids.

Collagen	Peak 1		Peak 2	
	*T*_max_ (°C)	Δ*H* (J/g)	*T*_max_ (°C)	Δ*H* (J/g)
CoFC	n.d.	n.d.	n.d.	n.d
AaEC	89.04	294.5	212.11	4.69
LaEC	83.30	241.8	174.35	5.86
CaEC	77.92	169.0	180.42	91.18

**Notes.**

CoFC commercial fish collagen hydrolysate AaEC acetic acid-extracted collagen LaEC lactic acid-extracted collagen CaEC citric acid-extracted collagen n.d. not detected

### Solubility of collagens

**Figure 7:**
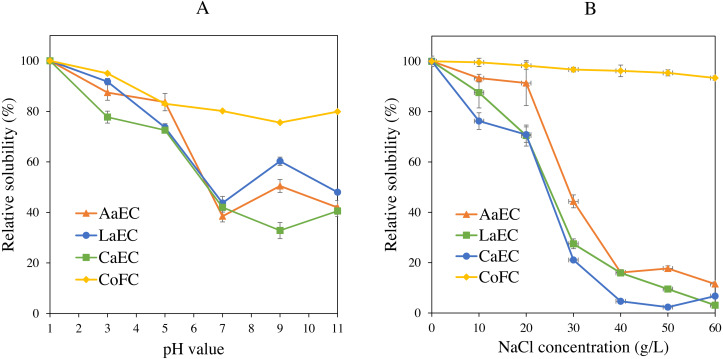
Solubility of lizardfish bone collagens extracted with different acids and commercial fish collagen hydrolysate. (A) At different pH levels and (B) after NaCl treatment. CoFC, Commercial fish collagen hydrolysate; AaEC, acetic acid-extracted collagen; LaEC, lactic acid-extracted collagen; CaEC, citric acid-extracted collagen.

The effect of lyophilized collagens and commercial fish collagen hydrolysates treated at different pH levels is shown in Fig. 7A. In general, the extracted collagens were soluble in acidic pH between 1 and 5, with a relative solubility of more than 70%, while CoFC sample was soluble in all pH treatment (>75%). The highest solubility (*P* < 0.05) for all collagen samples was observed at pH 1. The lowest relative solubility (*P* < 0.05) for AaEC and LaEC was at neutral pH and for CaEC was at alkaline condition (pH 9). As depicted in Fig. 7B. AaEC, LaEC and CaEC had similar solubility patterns (*P* < 0.05). Higher solubility was exhibited at low NaCl concentrations ranging between 0 g/L and 20 g/L for all extracted collagens but at all NaCl treatments for the CoFC sample showed higher solubility (>90%). Meanwhile, the solubility sharply decreased (*P* < 0.05) for lizardfish bone collagens with the addition of more than 30 g/L NaCl.

## Discussion

The high percentage of by-products generated from seafood processing drove us to investigate fish collagen that could be applied in the food and beverage, cosmetic, pharmaceutical and/or biomedical industries (Hashim et al., 2015). To this aim, we extracted collagens from lizardfish bone using different acids (*i.e.,* acetic acid, lactic acid and citric acid) and evaluated their biochemical characteristics, including Hyp, solubility, protein profile, UV absorption, X-ray diffraction, ATR-FTIR and thermal stability. As presented in Table 1, the yields of the extracted collagen ranged from 1.73% to 2.59% (*P* < 0.05) and were greater in comparison with previous studies on fishbone collagens from bigeye snapper (1.6%) (Kittiphattanabawon et al., 2005), carp (1.06%), grass carp (0.7%) (Wang et al., 2014), tilapia (0.5%) (Liu & Huang, 2016), bigeye tuna (0.1%) (Ahmed, Verma & Patel, 2020) and golden pompano (1.25%) (Cao et al., 2019). It can be assumed that the source of material (fish species) used is of greater importance for yielding fish collagen. In addition, different acids and extraction process applied during collagen production might be affected on the yield of all collagen samples (Ahmed, Haq & Chun, 2019). For Hyp content, AaEC showed the highest content, although it was not significantly different (*P* ≥ 0.05) from both LaEC and CaEC. The contents of Hyp from other fish collagens varied, as reported for tilapia (76-80 mg/g) (Liu & Huang, 2016), bigeye tuna (82–87 mg/g) (Ahmed, Verma & Patel, 2020), cobia (84–99 mg/g) (Zeng et al., 2012) and marine eel-fish (94–98 mg/g) (Veeruraj, Arumugam & Balasubramania, 2013). In the context of total collagen, the higher the Hyp content in the extracted samples, the higher the total collagen amount. The differences in Hyp and total collagen contents were affected by several factors, including species, size, age, structure and composition of fish tissue, as well as the extraction procedures (Regenstein & Zhou, 2007). All the acid-soluble collagens (ASCs) possessed significantly high L* values (*P* < 0.05), meaning that the extracted collagens were brighter than that of commercial fish collagen (CoFC). This is an important feature, as brighter collagen is preferable to processors in the development of new food products because there will be less or no interface with the product’s original color (Hofman et al., 2011; Gaurav, Nidheesh & Suresh, 2015). In comparison with barramundi skin collagen (44.76–65.41), the L* values of lizardfish collagens (88.00–88.54) were higher. In line with the L* value, the whiteness index (WI) observed in all AECs was also higher compared to that of CoFC, and AaEC was being the highest. It can be concluded that the acetic-aided process could provide the desirable color attributes of collagen from fishbones.

The ASCs extracted from the lizardfish bone were classified as type I collagen after confirmation *via* SDS-PAGE analysis, in which type I collagen has two identical subunits of alpha 1 (α_1_) and one subunit of alpha 2 (α_2_). This type I collagen has been widely studied in grass carp bone (Wang et al., 2014), seabass scale (Chuaychan, Benjakul & Kishimura, 2015), puffer fish skin (Iswariya, Velswamy & Uma, 2018), and sturgeon fish skin (Atef et al., 2020). Furthermore, there were no differences in the electrophoretic patterns after adding β-ME (reducing) and without adding β-ME (nonreducing), reflecting that the protein patterns of lizardfish bone collagens did not contain interchain disulfide bonds. These results were also in agreement with studies from golden goatfish scale collagen (Matmaroh et al., 2011) and bigeye tuna bone, skin and scale collagens (Ahmed, Verma & Patel, 2020). Moreover, the band intensity observed in the CaEC sample was not greater than that found in other collagen samples, and some minor bands appeared below 100 kDa. The reason might be due to the degradation of protein harbored in the citric-extracted collagen during the extraction process. Gustavson (1956) stated that a weak organic acid (citric acid) leads to hydrotropic swelling, which can replace hydrogen bonds of collagen with a hydrotropic substance, resulting in the removal of hydrophobic bonds and causing collagen degradation. For commercial fish collagen hydrolysate sample, on the other hand, no bands appear both treated with and without β-ME in the acrylamide gel. It might be due to the sample used in this study is hydrolysate type which contains small peptides with low molecular weight (León-López et al., 2019).

All the type I collagens from lizardfish bone had a maximum UV absorption at approximately 231 nm, indicating that the -COOH and -CO-NH_2_- components, as major functional groups investigated in collagen, tend to absorb UV light (Edwards & O’Brien, 1980). These results were in agreement with the collagens from catfish skin (232 nm) (Liu, Li & Guo, 2007), barramundi skin (230.3 nm), tilapia skin (230.9 nm) (Liao et al., 2018) and black ruff skin (232 nm) (Bhuimbar, Bhagwat & Dandge, 2019). In addition, collagen contains a low concentration of chromophore amino acids, such as histidine, phenylalanine, tryptophan, and tyrosine, in which those amino acids typically absorb UV light at 250 nm and 280 nm (Liao et al., 2018). In terms of FT-IR spectra evaluation, the ASCs extracted in this study were characterized by the presence of amide A, amide B, amide I, amide II and amide III, as shown in Fig. 5. To analyze the structure of lizardfish bone collagens based on the absorption regions (amides I–III), the difference in wavenumber (cm^−1^) between amides I and II can be determined using Δ*v* (*v*_I_–*v*_II_), where values <100 cm^−1^ indicate that the triple helical structure of collagen has been maintained (Nikoo et al., 2013). The results confirmed that all extracted collagens contained triple helical structures, with Δ*v* values of AaEC, LaEC and CaEC of 93.2, 98.8 and 95.1 cm^−1^, respectively. For commercial fish collagen (CoFC), however, the Δ*v* value was approximately 104.4 cm^−1^, indicating the presence of denatured collagen. Moreover, triple helical collagen can be determined using the absorption ratio (>1.0) of the amide III to the 1,450 cm^−1^ band (AIII/A1450) (Plepis, Goissis & DasGupta, 1996). After verification, all lizardfish collagen samples harbored triple helical structures because their absorption ratio values (AaEC = 1.4, LaEC = 1.3 and CaEC = 1.6) were greater than 1.0, while the absorption ratio of commercial fish collagen was lower than 1.0. This might be because the collagen reference used in this study was denatured. All the absorption peaks found in the lizardfish bone collagens were in accordance with previous studies on bigeye tuna collagen (Ahmed, Verma & Patel, 2020), tilapia skin collagen (Chen et al., 2016) and southern rays bream skin collagen (Sionkowska et al., 2015). Furthermore, the XRD diagram results confirmed that all the extracted collagens exhibited three different diffraction peaks, namely, A1, A2 and A3, except found in the CoFC samples (Fig. 6). A1 (7.04°–7.41°) is closely associated with the distance between the molecular chains of collagen fibrils (Marie-Madeleine et al., 2000). This peak, especially for the CaEC sample, was characterized by the highest d value than the LaEC and AaEC samples. A2 (19.54°–21.32°) represents the diffuse scattering caused by many structural layers of collagen fibrils, and A3 (31.70°–31.74°) describes the distance between adjacent amino acid residues along the central axis of its triple helical structure. From these results, the diffraction peaks found in the lizardfish collagen samples are typically similar to the XRD pattern observed in the tilapia skin and scale collagens (Chen et al., 2016). In the case of CoFC sample, only single peak appears in the XRD diagram. This result might be due to the degradation of molecular chains of collagen fibrils.

Regarding thermal stability, all ASC (AaEC, LaEC and CaEC) samples had two different maximum transition points (*T*_max_). The first point was related to the thermal denaturation of collagen and the water bonded molecules, while the second point might be characterized by the level of hydration and the properties of covalent cross-links. The highest value of extracted collagens at the first peak point was found in the AaEC sample (89.04 °C), followed by LaEC (83.30 °C) and CaEC (77.92 °C). The higher *T*_max_ indicated that the helical structure of all collagens was still maintained under thermal conditions, particularly in the AaEC sample. The reason might be due to the high content of hydroxyproline (Hyp), as stated by Matmaroh et al. (2011). The thermal stability of triple helical collagen was formed by pyrrolidine rings and hydrogen (H) bonding *via* the hydroxyl group of Hyp. For the enthalpy of denaturation (Δ*H*), the widest area under the peaks was detected in the AaEC samples (294.5 J/g), indicating the highest energy required to uncouple the α-chains of acetic-extracted collagen and convert them into random coils, compared to those of lactic and citric-extracted collagens. In comparison with previous studies from puffer fish (*Lagocephalus inermis*) (Iswariya, Velswamy & Uma, 2018), Pacific pomfret (*Brama australis*) (Sionkowska et al., 2015), and blue shark (*Prionace glauca*) (Elango et al., 2018) collagens, the acetic acid-extracted collagen from lizardfish bone had slightly greater *T*_max_ values. For CoFC sample, no peak detected in the DSC graph and it might be due to the removal of telopeptides region during hydrolysis by using proteolytic enzymes would produce unstable thermal structural that can lead to lower denaturation temperature of commercial fish collagen hydrolysate (Nalinanon et al., 2007). However, the difference in the thermal stability of fish collagen depends on the extraction process, imino acid composition and other living environmental factors (habitat and temperature) (Kittiphattanabawon et al., 2005; Iswariya, Velswamy & Uma, 2018).

Solubility studies showed that the ASCs were highly soluble under acidic conditions (pH 1.0 to pH 5.0). This finding was in accordance with collagen isolated from the skin of bigeye snapper (Nalinanon et al., 2007) and the skin, scale and bone of bigeye tuna (Ahmed, Verma & Patel, 2020). In contrast, at neutral pH and alkaline pH, the solubility of all collagens was reduced, as also observed in previous literature. Jongjareonrak et al. (2005) revealed that the loss of solubility at a particular pH might be due to an increase in hydrophobic-hydrophobic interactions among collagen molecules, and the total net charge becomes zero, particularly at the isoelectric point (pI). However, at pH 9 (alkaline condition), the solubility of AaEC and LaEC was slightly increased in the samples. The reason might be due to the effect of electrostatic repulsion between collagen molecules and hydration of charged residues above the pI (Chen et al., 2019; Kristinsson & Hultin, 2003). For the control sample (CoFC), higher solubilization point (more than 75%) found in all pH treatments (1-11). The increased solubility observed in the CoFC sample would be due to the smaller peptides and to exposure of their hydrophilic groups, which would increase interactions between the hydrophilic amino acids and water molecules (León-López et al., 2019). At the NaCl treatments, a higher solubility of ASCs was detected at low NaCl concentrations (up to 20 g/L), while the CoFC hydrolysate was highly soluble (>90%) in all NaCl treatment. Result of lizardfish bone collagens was in accordance with acid-solubilized collagen isolated from spotted golden goatfish scale (Matmaroh et al., 2011). However, the solubility of extracted samples sharply declined with high concentrations of NaCl (more than 30 g/L). This decrease in solubility might be due to a salting out effect during the precipitation process. When the salt concentration increased, the hydrophobic-hydrophobic interactions within polypeptide chains and competition for water with salt ions also increased and subsequently generated protein precipitation (Chen et al., 2016). From these results, lizardfish collagens can be a source of potential ingredients in food and pharmaceutical products.

## Conclusions

In this study, we investigated that the use of different acids could influence in the yield and characteristics of collagen derived from lizardfish bone. Yield of collagens varied with the highest was found in the citric acid-extracted collagen sample. However, the content of hydroxyproline and profile of protein pattern and thermal stability presented that the acetic acid-extracted collagen has better characteristics compared to those of citric and lactic-extracted samples. Taken together, lizardfish bone collagens may be used as alternative collagens for further application in industrial processes, and more specifically, acetic acid-extracted collagen (AaEC) would be considered more effective in terms of the characteristics evaluated in this study.

## Supplemental Information

10.7717/peerj.13103/supp-1 Table S1 Raw data of Color attributes (*L* *, *a* *, *b* * and whiteness index) of collagens from lizardfish bones extracted with different acids

10.7717/peerj.13103/supp-2 Figure S1 Raw data of Yield, hydroxyproline (Hyp) and collagen content of lizardfish bone collagen extracted with different acids

10.7717/peerj.13103/supp-3 Figure S2 Raw data of UV absorption of lizardfish bone collagens extracted with different acids

10.7717/peerj.13103/supp-4 Figure S3 Raw data of FTIR patterns of lizardfish bone collagen extracted with different acids

10.7717/peerj.13103/supp-5 Figure S4 Raw data of X-ray diffraction diagram of lizardfish bone collagens extracted with different acids

10.7717/peerj.13103/supp-6 Figure S5 Raw data of Solubility of lizardfish bone collagens extracted with different acids

## Additional Information and Declarations

### Data Availability

The following information was supplied regarding data availability: The raw measurements are available in the Supplementary Files.
